# Supplementary material for: Fabrication and Characterization of Tedizolid Phosphate Nanocrystals for Topical Ocular Application: Improved Solubilization and In Vitro Drug Release
Source: Pharmaceutics. 2022 Jun 23;14(7):1328. doi: 10.3390/pharmaceutics14071328 (PMC9320520; doi:10.3390/pharmaceutics14071328)
Supplement: Supplementary file 1 [file pharmaceutics-14-01328-s001.zip › pharmaceutics-1771181-supplementary/pharmaceutics-1771181-supplementary.pdf]

Supplementary Materials

Analytical method for TZP assay

A slight modification in the existing HPLC-UV methods [1] was successfully employed to analyse TZP in the samples obtained during *in vitro* experiments. The calibration curve was obtained between the concentrations of TZP (μgmL^{-1}) and the area of corresponding peaks obtained by chromatographic analysis. The calibration curve of the drug was found linear in the mentioned concentration ranges (0.25-50 μgmL^{-1}) with co-efficient of correlation (r^2 = 0.9996). The straight-line equation obtained for the calibration data was $Y = 61528x - 6420$, where Y was the peak area for the drug and x was its concentration. The regression equation was found good to calculate the drug concentrations during *in vitro* experiments.

Supplementary Figure

Figure S1

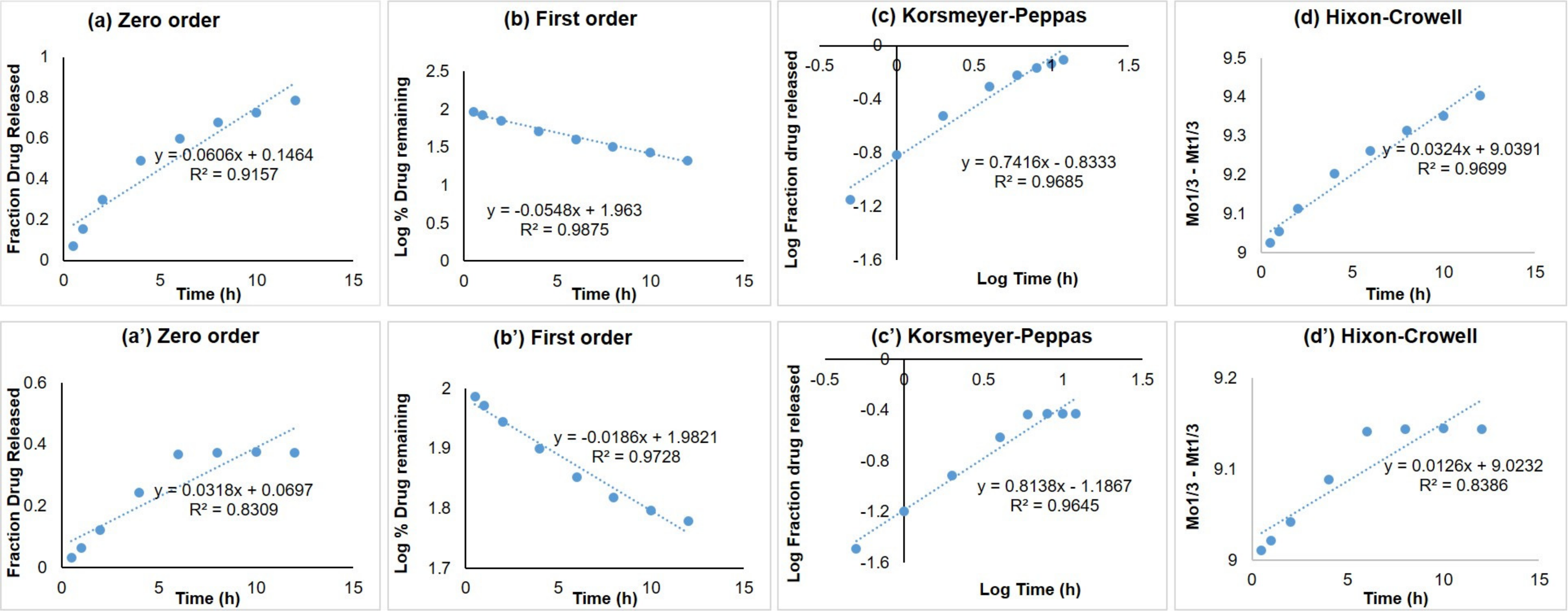

Figure S1. Different kinetic model plots for TZP-NC$_1$ (a, b, c and d) and the plots for conventional aqueous suspension of TZP-pure (a', b', c' and d')

References

1. Moaaz, E.M.; Abdel-Moety, E.M.; Rezk, M.R.; Fayed, A.S. Stability-Indicating Determination of Tedizolid Phosphate in the Presence of its Active Form and Possible Degradants. *J Chromatogr Sci* **2021**, 10.1093/chromsci/bmab045, doi:10.1093/chromsci/bmab045.
